# Supplementary material for: The Role of ZEB2 in Human CD8 T Lymphocytes: Clinical and Cellular Immune Profiling in Mowat–Wilson Syndrome
Source: Int J Mol Sci. 2021 May 18;22(10):5324. doi: 10.3390/ijms22105324 (PMC8158478; doi:10.3390/ijms22105324)
Supplement: Supplementary file 1 [file ijms-22-05324-s001.zip › ijms-1203178-final-suppl.pdf]

Supplementary Table S1: Baseline vaccine responses to Diphtheria, Tetanus and *Haemophilus influenza* type b

	Gender	Age (years)	*Anti-Diptheria (IU/mL)	~Anti-Tetanus (IU/mL)	∞Anti-H.influenzae type b (mcg/mL)
**1**	**M**	**22**	**0.04**	**0.36**	**0.16**
**2**	**M**	**21**	**0.42**	**0.18**	**0.12**
**3**	**M**	**20**	**0.26**	**0.81**	**0.14**
**4**	**F**	**12**	**0.03**	**<0.01**	**0.25**
**5**	**M**	**11**	**0.34**	**0.14**	**ND**
**6**	**M**	**3**	**0.08**	**0.25**	**0.71**

*Diptheria: 0.01-0.1IU/mL seropositive (but may not be fully protective), >0.1IU/mL protective

~Tetanus: ≥0.16IU/mL protective

∞H. influenza type B: >0.15mcg/mL equates to short term, >1.0 mcg/mL equates to long term protection

Supplementary Table S2: Pneumococcal IgG responses pre- and post-unconjugated pneumococcal vaccine (Pneumovax 23)

	Age (y)	Baseline Pneumococcal serotype specific IgG (ug/mL)							Pneumovax 23* given (Yes/No)	~Pneumococcal serotype specific IgG post polysaccharide vaccination (ug/mL)						
		2	8	10A	11A	15	17F	33F		2	8	10A	11A	15	17F	33F
1	22	1.5	0.3	>10.1	<0.1	0.6	0.2	0.4	Y	14.9	>14.2	>13	2.8	8.8	3.6	3.1
2	21	0.2	0.2	0.1	0.1	0.2	<0.1	1.1	N	-	-	-	-	-	-	-
3	20	-	1.1	1.5	1.1	0.6	>4.9	1.1	N	-	-	-	-	-	-	-
4	12	-	0.8	0.9	0.4	0.3	1.1	0.7	N	-	-	-	-	-	-	-
5	11	-	0.1	0.2	0.1	0.2	0.3	0.2	N	-	-	-	-	-	-	-
6	3	-	0.9	0.2	<0.1	0.1	0.3	<0.1	Y	>8.6	>3.3	0.7	>5.3	4.2	>3.9	>5

*Pneumovax 23 contains serotypes 4, 6B, 9V, 14, 18C, 19F, 23F, 2, 8, 10A, 11A, 15B, 17F, 20 and 33F.

~pre and post samples required for interpretation; responses considered protective if post vaccination responses >1.0mcg/mL, or >2-fold increase

Supplementary Table S3 – Monoclonal antibodies used in this study

Antibody	Fluorochrome	Company
CD3	PerCP-Cy5.5	Becton Dickinson
CD4	AlexaFluor 700	Becton Dickinson
CD4	PE	Becton Dickinson
CD8	APC-Cy7	Becton Dickinson
CD8	BV711	Becton Dickinson
CD16	BV421	Becton Dickinson
CD19	BV786	Becton Dickinson
CD25	BB515	Becton Dickinson
CD25	APC	Becton Dickinson
CD28	APC	Becton Dickinson
CD33	PE-Cy7	Becton Dickinson
CD38	PE-Cy7	Becton Dickinson
CD45	AlexaFluor 700	Becton Dickinson
CD45-RA	BV605	Biolegend
CD45-RO	FITC	Becton Dickinson
CD49d	BV510	Biolegend
CD56	APC	Becton Dickinson
CD62L	APC-eFluor780	eBioscience
CD127	PE-CF594	Becton Dickinson
CD127	BV786	Becton Dickinson
CD134	PE	Becton Dickinson
CD161	PE-Cy7	Biolegend
CCR6	BV421	Biolegend
CX3CR1	FITC	MBL
Granzyme B	APC	Invitrogen
Integrin Beta-7	APC	Becton Dickinson
PD-1	BV421	Becton Dickinson
Perforin	PE	Biolegend

Supplementary Figure S1:

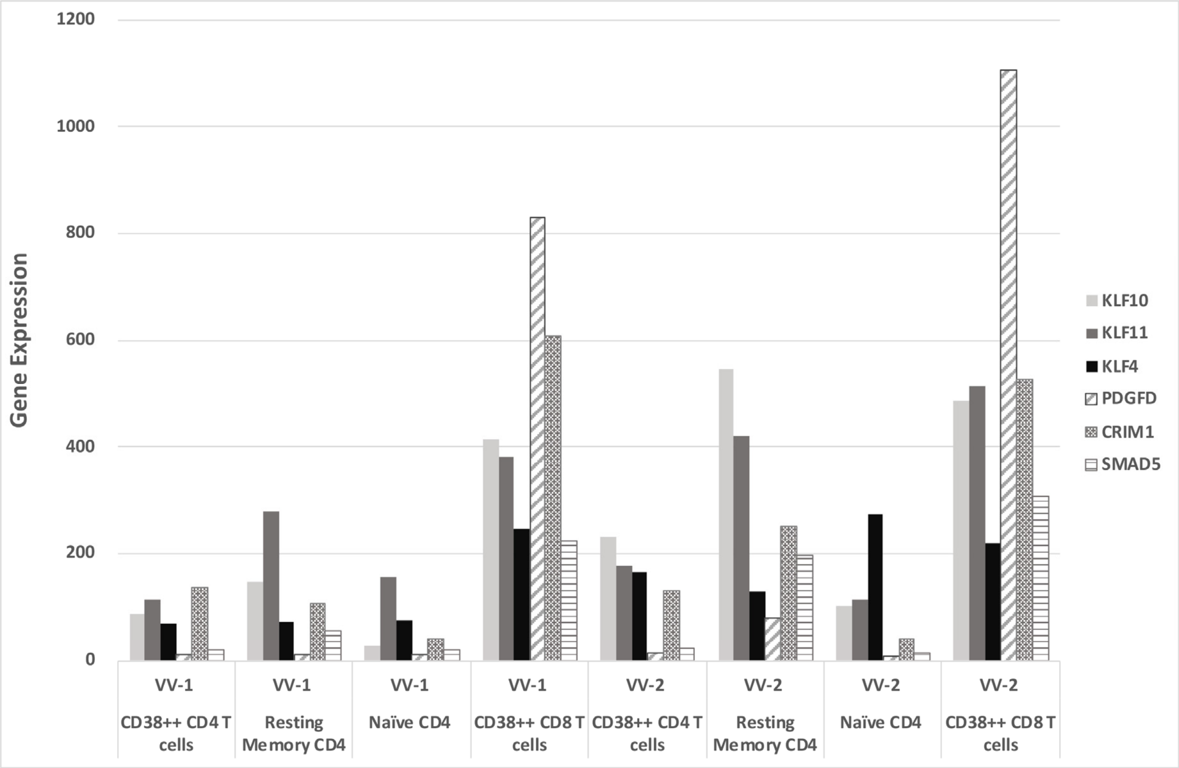

Supplementary Figure S1. Gene expression profiling of KLF10, KLF11, KLF4, PDGFD, CRIM1 and SMAD5 RNA, in microarrays of early-activated effector CD38++ CD8 T cells following vaccinia inoculation. Four purified T cells subsets were cell sorted from fresh PBMC: CD38++ CD4 T cells; resting memory CD4 T cells; naïve CD4 T cells; and CD38++ CD8 T cells from each of 2 healthy adult donors, VV1 and VV2. RNA levels for each subset were normalized and calculated in Affymetrix MAS 5.0 software.

Supplementary Figure S2:

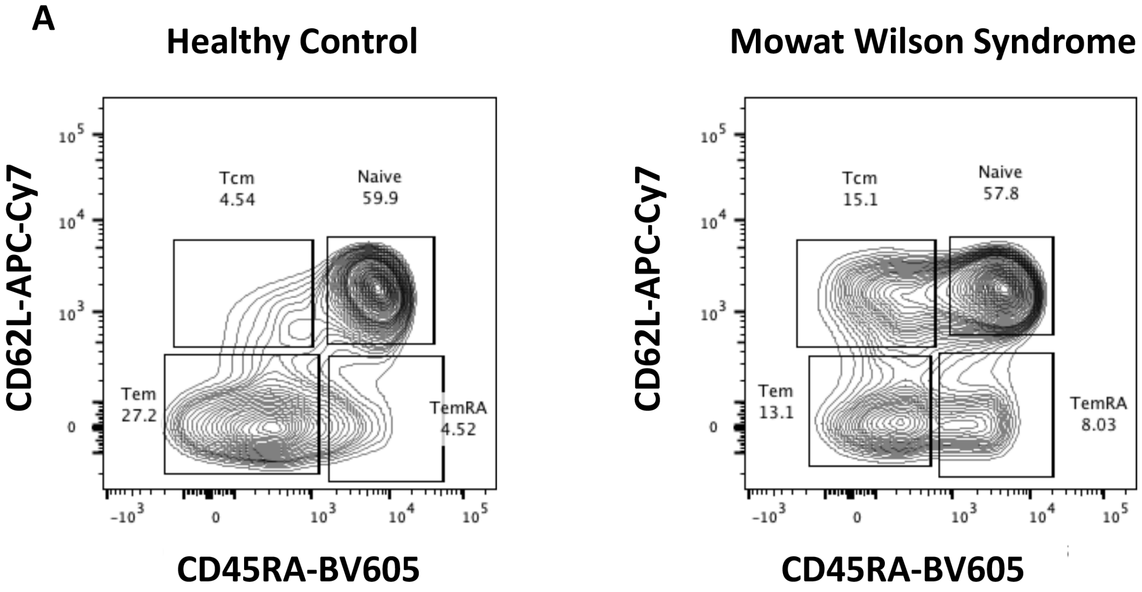

B

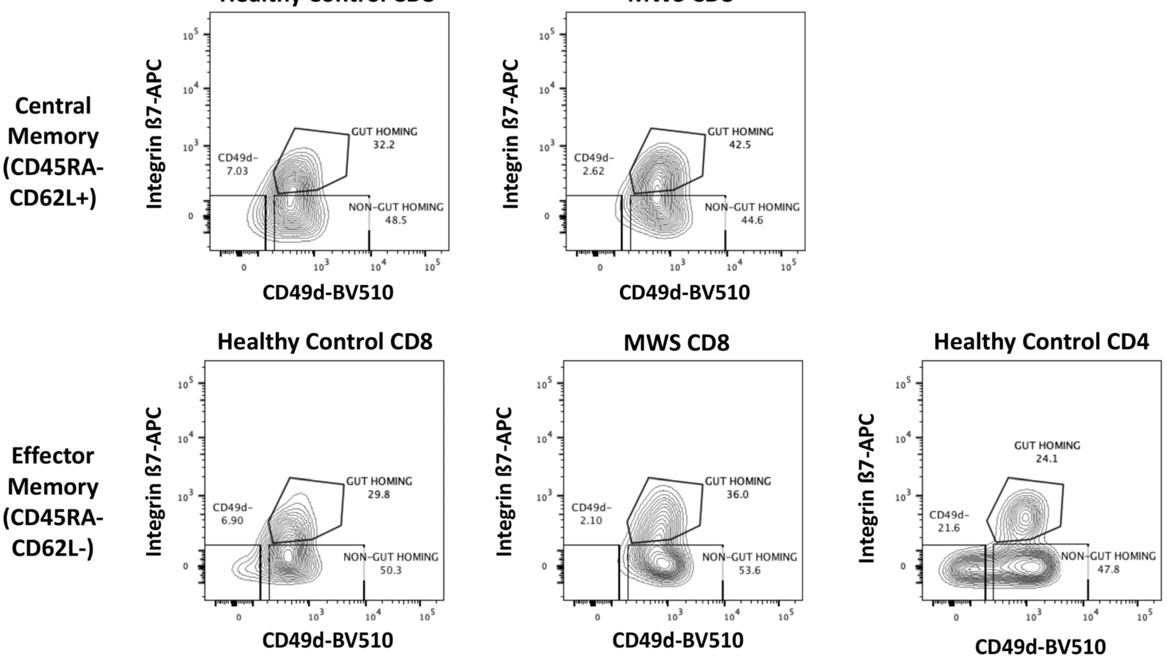

Healthy Control CD8
MWS CD8
Healthy Control CD4
Central Memory (CD45RA- CD62L+)
Effector Memory (CD45RA- CD62L-)
Integrin ß7-APC
CD49d-BV510
GUT HOMING 32.2
CD49d− 7.03
NON−GUT HOMING 48.5
GUT HOMING 42.5
CD49d− 2.62
NON−GUT HOMING 44.6
GUT HOMING 29.8
CD49d− 6.90
NON−GUT HOMING 50.3
GUT HOMING 36.0
CD49d− 2.10
NON−GUT HOMING 53.6
GUT HOMING 24.1
CD49d− 21.6
NON−GUT HOMING 47.8

C

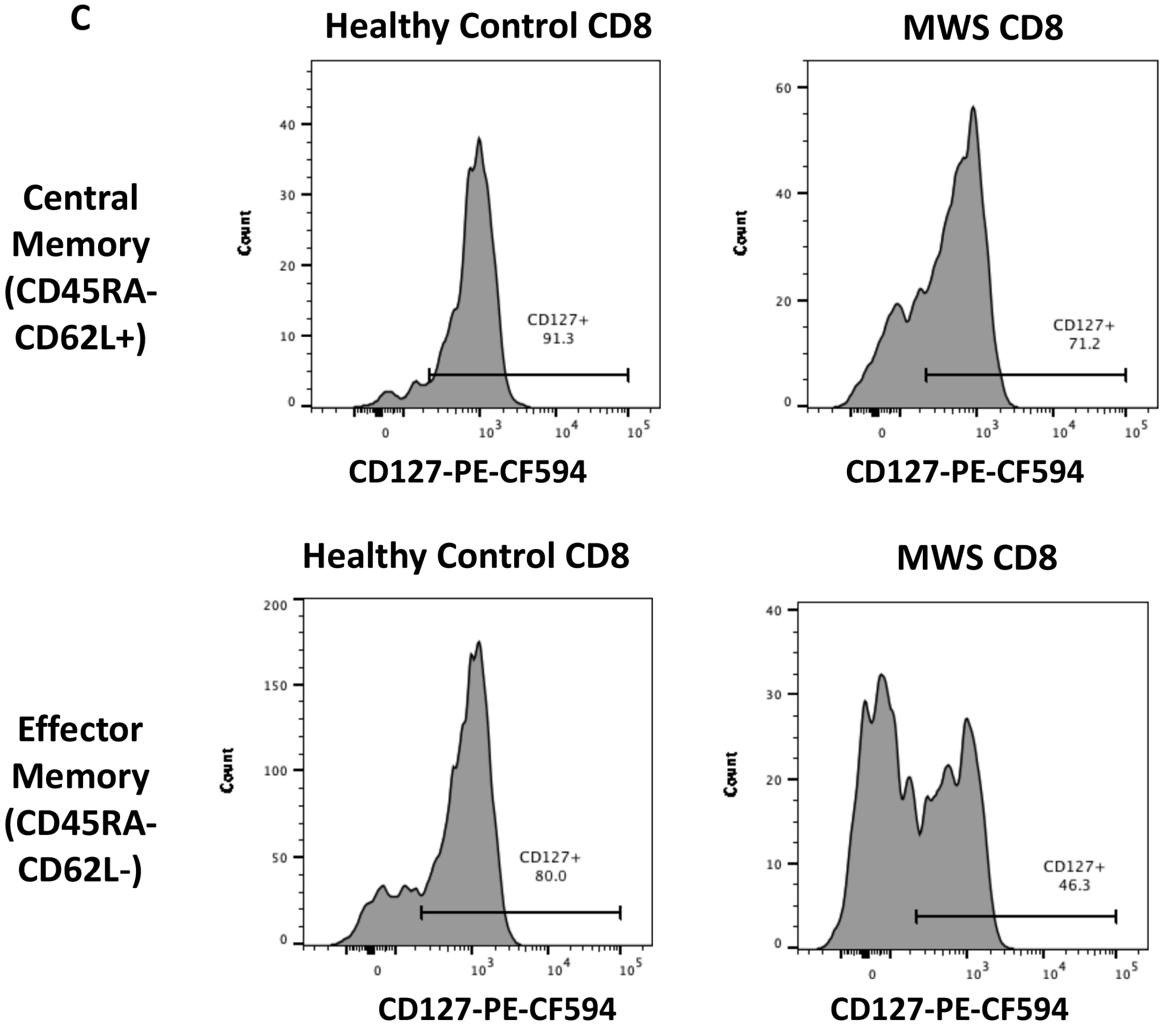

Healthy Control CD8
MWS CD8
Central Memory (CD45RA- CD62L+)
Effector Memory (CD45RA- CD62L-)
Count
CD127-PE-CF594
CD127+ 91.3
CD127+ 71.2
CD127+ 80.0
CD127+ 46.3

D

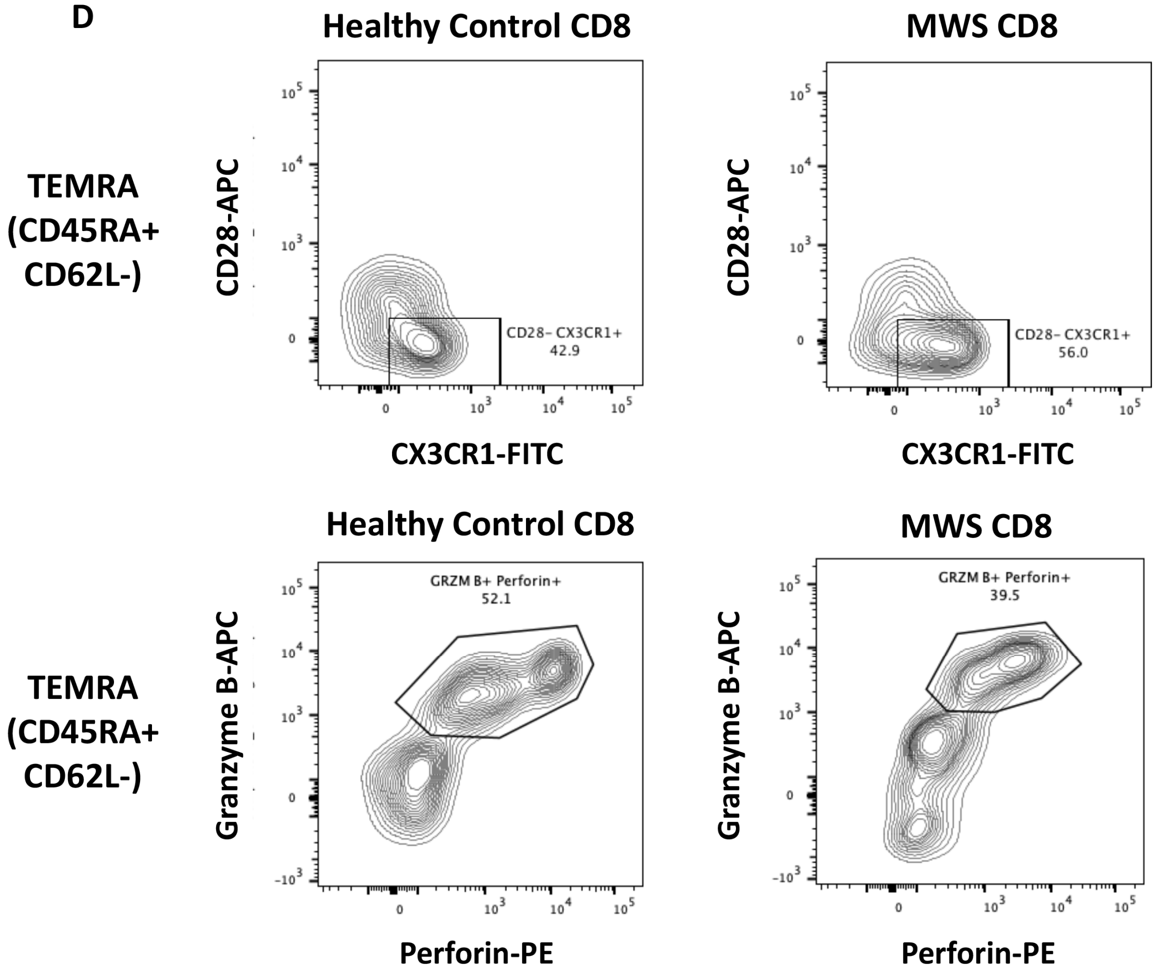

E

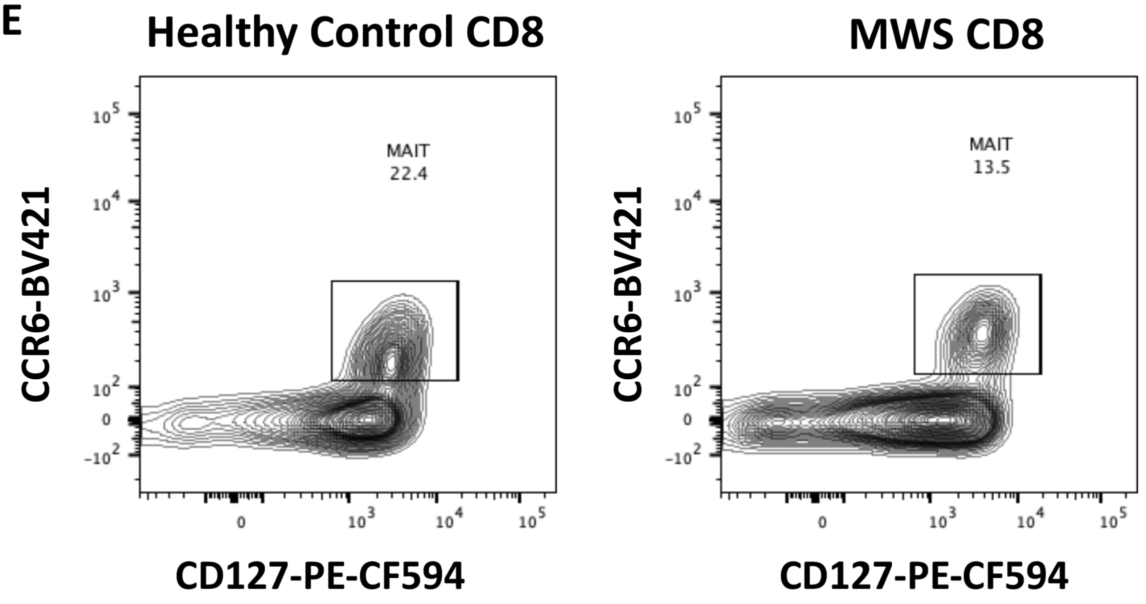

Supplementary Figure S2: Representative flow plots showing gating strategy for CD8 subsets in healthy controls and Mowat Wilson Syndrome (MWS) patient samples, respectively. (A) Naïve CD8 T cells (CD45RA+CD62L+), Central Memory (TCM) cells (CD45RA-CD62L+), Effector Memory (TEM) cells (CD45RA-

CD62L-) and Terminally Differentiated Memory (TEMRA) cells (CD45RA+CD62L-). (B) Upper row: Central memory CD8 T cells gut-homing memory cells (Integrin ß7+CD49d+), Non-gut homing memory cells (Integrin ß7-CD49d+) and CD49d-negative cells. Lower row: Effector memory CD8 T cells gut-homing memory cells (Integrin ß7+CD49d+), Non-gut homing memory cells (Integrin ß7-CD49d+) and CD49d-negative cells. On far right, for reference to show how gates were set, effector memory CD4 T cells with the same populations. (C) Upper row: Central memory CD8 T cells CD127+ cells. Lower row: Effector memory CD8 T cells CD127+ cells. (D) Upper row: TEMRA CD8 T cells (CD45RA+CD62L-) showing loss of CD28 and gain of CX3CR1. Lower row: TEMRA CD8 T cells (CD45RA+CD62L-) showing dual expression of Granzyme B and Perforin. (E) MAIT memory (CD45RA-) CD8 T cells gated on CCR6+CD161^{high}CD127^{high}.
